# Was a forced lockdown adequate for a country in conflict? A psychological perspective from the Syrian population

**DOI:** 10.5339/qmj.2021.17

**Published:** 2021-05-03

**Authors:** Ameer Kakaje*, Sabina Mansour, Amjad Ghareeb

**Affiliations:** Faculty of Medicine, Damascus University, Damascus, Syria E-mail: ameer.kakaje@hotmail.com

**Keywords:** Syria, developing country, COVID-19, lockdown, mental health, anger, stress, depression, anxiety, healthcare workers, conflict.

## Abstract

Background: Syria has suffered for nine years from a conflict that left over 11.1 million inhabitants in need of humanitarian assistance and over 80% in poverty. A ten-week-long full lockdown was enforced in Syria and successfully minimized the spread of COVID-19. This study aims to estimate the occurrence of mental health disorders after lockdown termination among the citizens of war-torn Syria.

Methods: Online questionnaires, which included demographic and war-related questions, Dimensions of Anger Reactions 5 (DAR-5) and the Depression Anxiety Stress Scale-21 (DASS-21) were distributed to different social media groups.

Results: This study recruited 1445 participants, of which 515 (35.6%) were males, the mean age was 24.8 ± 6.3 years, 38% had problematic anger, 64% had moderate to very severe depression, 42.9% had moderate to severe anxiety and 39.7% had moderate to severe stress. Increased living expenses, not being able to go out and a reduced ability to earn income and provide food were significantly associated with the psychological burden after the lockdown (*p* < 0.05). The association of war variables with mental disorders was weaker than the effect of the deteriorating economy. Other healthcare workers had more severe distress than doctors, who themselves were found to have less distress than the general population (*p* < 0.05). Anger scores were approximately equal, regardless of the type of work. Finally, shisha smoking was associated with worse mental health (*p* < 0.05).

Conclusion: The psychological burden of the damaged economy surpassed the direct damage due to COVID-19 and the effect of years of conflict. Urgent interventions are required, as this burden may continue for years, if not for decades. A full lockdown in countries with fragile economies may delay the spread of the virus, but it will severely damage the economy, which will lead to a deterioration of the mental health of their citizens.

## Introduction

Syria has been in conflict since 2011, which left the population in great distress as more than 11.1 million people needed humanitarian assistance, 5.5 million fled the country and 6 million were internally displaced. This left more than 80% of the population of Syria living below the poverty line^1^. A forced lockdown was introduced in Syria to prevent a COVID-19 outbreak, despite the absence of confirmed cases. All employees were ordered to stay at home unless they worked in a medical or essential field, and most shops were closed unless they provided essential goods. This lockdown lasted approximately 10 weeks and was a preventive measure, as Syria did not have the proper means to react to COVID-19 if many people became infected. There were fewer than 60 confirmed cases before lockdown termination on 26/05/2020. There was a dramatic increase in the case count in the subsequent three weeks, and it reached around 200 confirmed cases on 19/06/2020^2^. This high incidence of cases was mainly due to Syrians having to leave home to work and obtain food, which made them crowd together after the lockdown. Subsequently, the psychological burden became very high, mainly from the destroyed economy.

After the lockdown, all essential and non-essential goods at least doubled or tripled in price. This caused people to panic as household and living expenses, including food and medications, became very high despite their incomes remaining the same. People were now suffering from a shortage of medication and food, and no practical solution was available. Previous outbreaks had mainly been studied for their physical health consequences, while their long-term effects on mental health were neglected. However, all disasters, either natural or man-made, affect the social structure and have a strong impact on mental health^3^.

Presently, the international community is making the same mistakes as they did with the Ebola outbreak: their involvement in vulnerable countries, if any, is insufficient. The effects of said outbreak reached beyond the infection itself to the education and health sectors, and it halted any economic progression^4^. Another study observed that indirect mortality from Ebola was as important as direct mortality, if not greater^5,6^.

Post-traumatic stress disorder (PTSD) and anger were among the most common mental health issues experienced during quarantine^7^. Moreover, infection fears, anxiety, frustration, disabling loneliness, financial loss and boredom were among the major stressors^7,8^. Many countries, particularly low-income ones, faced major problems in research during COVID-19; even programs of high priority, such as those addressing tuberculosis, which is endemic in Syria, were affected^9^. Healthcare providers in particular were affected as they were directly facing COVID-19, which further increased their distress. In addition, Syria is considered one of the most fragile countries and on very high alert worldwide^10^. It is a low-income country and thus highly vulnerable to public health crises^9^. Furthermore, the prevalence of mental health disorders in Syria is one of the highest in the world^11^. This study estimates the effect of lockdown on psychological aspects in war-torn Syria two weeks after the termination of lockdown and the reopening of restaurants and cafes.

## Methods

### Sampling

This was a cross-sectional study conducted across Syria, covering the period from 08/06/2020 to 17/06/2020. Online Arabic surveys were used and filled in by participants living in Syrian governorates. Participants living in other countries were not included. The surveys were distributed multiple times in more than 40 online social media groups and pages that covered several topics. These online social groups included various topics, such as science, debates, cuisine, entertainment, university-related discussions, and others, so this method should provide little bias. Google forms were used on social media websites such as Facebook. This method allowed us to distribute surveys across the country in a short time and reach a large population without requiring funding for the research. This also minimized the risk of data collectors acquiring COVID-19.

The targeted population was all participants who lived in Syria during the war and were in Syria during the 10-week lockdown. We could not target people who did not have internet access at that time or were not interested in the survey.

### Consent and approval for the study

Informed consent that ensured anonymity and confidentiality was obtained before proceeding with the survey. Informed consent was also given for the use and publication of the data without any personal identifiers.

The ethical aspects of the study were approved by Damascus University deanship in Damascus, Syria. The approval reference number is 170453.

### Questionnaires

#### Socioeconomic status (SES)

It is not socially acceptable to ask about income in Syria, and there is no valid method to determine SES levels according to income in Syrian pounds, particularly because their value is changing rapidly. Therefore, SES was estimated by asking about the individual’s living situation (own, rent, live with a friend, or live in government housing) and educational level. We also assessed household income as adequate or inadequate for obtaining essential goods. We could not use any validated methods for SES assessment as none of them have been validated in Syria.

#### Screening for depression, anxiety, and stress

An Arabic version of the Depression Anxiety Stress Scale-21 (DASS-21), which is a probabilistic but not a deterministic measure of distress, was used. Scores were used to determine which groups were in greater distress^12^. The English norms of the DASS-21 were appropriate for the Arabic version, and the results supported the cross-cultural universality^13^. The DASS-21 has three subscales: depression, anxiety, and stress. It is meant to minimize the overlap between depression and anxiety^14^. Each question’s score ranges from 0 to 3, and each of the three subscales has 7 questions. The sum of scores on these 7 questions for each subscale is then multiplied by 2, so that the maximum score for each subscale is 42, and the total possible score on the DASS-21 is 126. Each subscale has its own score interpretation. For depression, scores of (0–4), (5–6), (7–10), (11–13), and (14+) indicate normal, mild, moderate, severe, and extremely severe, respectively. For anxiety, scores of (0–3), (4–5), (6–7), (8–9), and (10+) indicate normal, mild, moderate, severe, and extremely severe, respectively. For stress, scores of (0–7), (8–9), (10–12), (13–16), and (17+) indicate normal, mild, moderate, severe, and extremely severe, respectively.

#### Anger measurement

The Dimensions of Anger Reactions 5 (DAR-5) scale is based on five items that assess anger in the last four weeks. The DAR-5 also assesses the effect of anger on social functioning, and it has been translated into Arabic^15–17^. A score greater than 12 is an indicator of problematic anger. A higher score is indicative of more symptoms.

#### COVID-19 questions

Some of the COVID-19 question ideas in our study were taken from The CoRonavIruS Health Impact Survey (CRISIS)^18^, and we added extra questions related to the current situation as seen in the tables.

#### Other questions

Basic demographic questions were also asked (Table 1).

#### Definitions

Consanguinity and work-type categories were defined according to a previous study on this topic^19^. Living expenses were defined as products purchased in shops, grocery items, and eating out, while household expenses were defined as bills and rent. Going out was defined as leaving the house for any reason. Regular cigarette smoking was defined as smoking every day of the week, regardless of the quantity. Regular shisha smoking was defined as smoking two or more times per week. A chronic medical condition was defined as any medical condition that required continuous monitoring and medication.

### Data analysis

IBM SPSS software, version 26 for Windows (IBM Corp.: Armonk, New York) was used for data analysis. One-way analysis of variance (ANOVA), linear regression and independent t-tests were performed. Pearson’s correlation was also calculated. Values of less than 0.05 for two-tailed *p* values were considered statistically significant.

## Results

**Figure 1.**
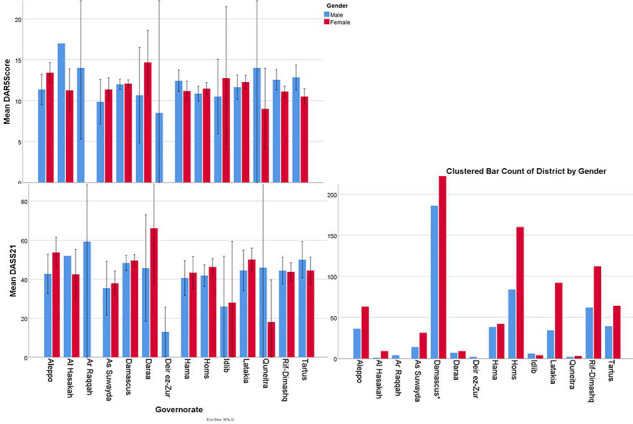
The distribution of DAR-5 and DASS-21 scores by governorate of residence^^*^^The number of females from Damascus in this study is 341.

**Figure 2.**
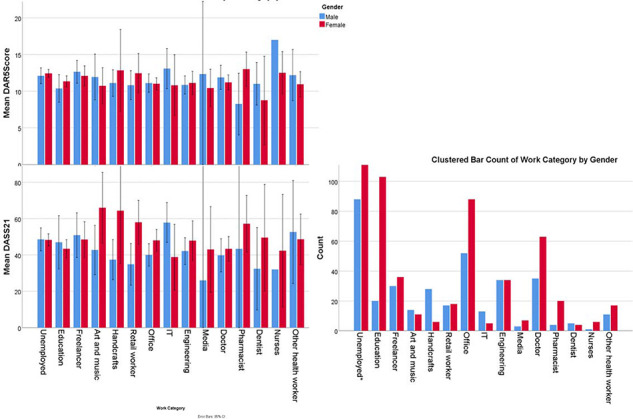
Work category according to anger and DASS-21 scores^^*^^The number of unemployed females in this study is 264.

This study recruited 1445 participants; 515 (35.6%) were male, and the overall mean age was 24.8 ± 6.3 years. Participant characteristics are summarized in Table 1. DASS-21 scores and governorates of current residence are listed in Figure 1. In the studied sample, 38% had problematic anger, 64% had moderate to very severe depression, approximately 42.9% had moderate to severe anxiety, and approximately 39.7% had moderate to severe stress. There were no differences in problematic anger between doctors (36.8%) and the whole sample (38%) (Figure 2). However, doctors had lower DASS-21 scores, compared with those of the whole sample (Table 1), as 58.3% of doctors had moderate to severe depression, approximately 35% had moderate to very severe anxiety, and 31% had moderate to very severe stress. Interestingly, although nurses had higher anger scores, they had lower DASS-21 scores. However, although pharmacists and other healthcare workers had DAR-5 scores that were not significantly different from those of doctors, they had higher depression, anxiety, and stress scores than doctors. DAR-5 and DASS-21 scores according to type of work are demonstrated in Figure 2. Only nine subjects were younger than 16 years, but none were younger than 14 years.

### Mental distress

According to Pearson’s correlation, older age was significantly and inversely correlated with anger, stress, depression, and anxiety scores (*p* < 0.01) and (r = − 0.138, r = − 0.093, r = − 0.146, r = − 0.077), respectively. We used independent t-tests and one-way ANOVA to demonstrate the differences in mean DAR-5 and DASS-21 scores according to different characteristics (Table 2 and Table 4). Interestingly, only anxiety scores were statistically significantly different between genders, with females having higher scores (*p* = 0.027). Living in a city was associated with higher stress and depression scores (*p* < 0.05). Participants with high school or university education had higher depression scores (*p* = 0.021). Although living in a rented house was not associated with higher DASS-21 or DAR-5 scores, a less adequate monthly income was correlated with a higher score (*p* < 0.05). Smoking was associated with higher scores on the DASS-21 and DAR-5, particularly regularly smoking cigarettes and smoking both cigarettes and shisha (*p* < 0.05).

We employed linear regression using anger, stress, depression, and anxiety as dependent variables. We included as independent variables the four questions about war, smoking, and monthly income adequacy. When regressed on the anger score, monthly income adequacy and smoking were significant at (*p* < 0.001), with R^2^ of 1.2% and 0.8%, respectively. When regressed on the depression score, only monthly income adequacy was significant (*p* < 0.001), and R^2^ was 1.3%. When regressed on the anxiety score, distress from war noise, changing place of residence due to war, and monthly income adequacy were significant (*p* < 0.05), with R^2^ of 0.9%, 0.6%, and 0.5%, respectively. When regressed on the stress score, monthly income adequacy and distress from war noise were significant (*p* < 0.05), with R^2^ of 0.9% and 0.6%, respectively.

### COVID-19 variables

COVID-19 questions and responses are demonstrated in Table 3, and differences were determined using one-way ANOVA (Table 4). A reduced ability to work was significantly associated with higher DAR-5, depression, anxiety, and stress scores (*p* < 0.05). Furthermore, stressors from all sources were associated with significantly higher scores (*p* < 0.001). Having difficulty purchasing any personal protective equipment was associated with significantly higher DAR-5 and DASS-21 scores, while having easy access to them was associated with lower scores (*p* < 0.05). However, anger, anxiety, stress, and depression were not correlated with taking more personal protective measures (*p*>0.05).

We used linear regression with anger, stress, depression, and anxiety as dependent variables. We included as independent variables the 10 questions regarding COVID-19 distress (from Table 4), reduced ability to work in the last two weeks, and the four questions regarding war variables. When regressing on DAR-5, household expenses, being able to go out, reduced ability to earn income and providing food were significant (*p* < 0.05), with R^2^ of 4.5%, 1.2%, 0.6%, and 0.4%, respectively. When regressing on depression scores, household expenses, living expenses, obtaining medications, reduced ability to earn money, work negatively being affected, and being distressed by war noise were significant (*p* < 0.05) with R^2^ of 6.1%, 1.2%, 0.8%. 0.4%, 0.4%, and 0.4%, respectively. When regressing on anxiety score, obtaining medications, being able to go out, changing place of living due to war and household expenses were significant (*p* < 0.05), with R^2^ of 5.1%, 0.8%, 0.6%, and 0.3%, respectively. When regressing on anxiety score, obtaining medications, being able to go out, changing place of residence, and household expenses were significant (*p* < 0.05), with R^2^ of 5.1%, 0.8%, 0.6%, and 0.3%, respectively.

## Discussion

### Anger, anxiety, depression, and stress

Before the COVID-19 outbreak, a study that used similar methods to ours and used similar online groups found that the prevalence of having two or more positive PTSD symptoms was 60.8%, and that of having a moderate to severe mental disorder was 61.2%, among adults^11^. Furthermore, approximately 53% of school students in Damascus, Syria had PTSD, 62% had problematic anger, and 61% had a moderate to severe mental health disorder^20^ . However, during the COVID-19 lockdown, another online study that used similar methods and online groups to this study found the prevalence of having two or more symptoms of PTSD was 42.7%, and that of a moderate to severe mental disorder was approximately 42.6%^19^. Although there were previous studies using the same methods, we could not determine the differences in participant groups, which might affect the comparisons.

A systematic review that included 15 different studies about Syrian refugees in 10 different countries found that 43% of refugees had PTSD, 40.9% had depression, and 26.6% had anxiety. It also found that Syrian refugees were 10-fold more prone to PTSD and other disorders than the general population^21^.

The prevalence of depression and anxiety in our study was significantly higher during the pandemic and after weeks of lockdown compared with during the lockdown and during the war. We speculate that the full lockdown and the economic collapse caused more severe psychological stress than the nine years of war. However, the prevalence of anger in our study was lower than that in the school study^20^. We speculate that the reaction of school students and adults to stress might be different, which justifies the lower prevalence in school students. Interestingly, no significant differences in problematic anger between males and females were found in our study, which was a similar result to that found in the previous study^20^.

Monthly income adequacy and regular smoking were the main factors associated with higher anger scores, apart from COVID-19 questions, which may reflect a reciprocal association between low SES and regular smoking with anger. Cigarette smoking was associated with anger features which were dependent on gender^22^. It was also found that heavy smokers had higher rates of anger, depression, and anxiety, while moderate cigarette smoking was associated with higher anger but not with the other negative emotions^22^. Anger, depression, anxiety, and stress were also significantly associated with regular smoking, and cigarette smoking was associated with higher mean scores than shisha smoking. We could not measure the amount smoked in this study, but responders who regularly smoked both shisha and cigarettes tend to be heavy smokers; therefore, we speculated that this was the reason for their higher overall scores. Smoking in Syria was also associated with laryngopharyngeal reflux and allergic rhinitis, and all previous factors were associated with war variables in Syria, mainly distress from war noise, all of which were found to significantly affect quality of life^^20,23–25^^.

Our study found that distress from war noise was associated with higher anxiety and stress scores. Age had a negative correlation with mental health in our study, in agreement with a previous study in Syria during the COVID-19 pandemic^19^ but in contrast to another one conducted in Syria before COVID-19^11^.

A low SES is, without a doubt, a major stressor, and our findings that not having an adequate monthly income is associated with more severe mental disorder were expected. However, unexpectedly, living in a rented or owned house was not significantly associated with anger or DASS-21 scores. We speculate that, as the general condition was deteriorating, the rent effect was neglected as other major stressors became increasingly prominent. Living with a chronic condition or with an individual who had a chronic condition was associated with higher anxiety and stress scores, which may explain the high distress over medication procurement. However, this association was not found with anger and depression scores.

## Covid-19

Mental health disorders were prevalent in the studied sample (Table 1). A study on Syrian schools conducted directly before COVID-19 lockdown in Syria confirmed that the prevalence of PTSD in Damascus was around 53%, that of moderate to severe mental health illness was 61%, and that of problematic anger was 62%^20^ . It was also found in a study approximately one year before COVID-19 that war variables were the major contributors to distress among adults in the Syrian population^11^. That study used similar methods and a similar target population to this study; therefore, it will be easier to draw comparisons with it. However, after four weeks of mandatory lockdown, using the same methods and targeted population, 42.6% of the population had a moderate to severe mental disorder^19^. Furthermore, that study demonstrated that participants experienced relationship deterioration, which in turn was associated with severe mental disorders and PTSD. Reduced ability to work and providing food were also major stressors^19^.

In our study, household expenses and a reduced ability to obtain medications were major stressors associated with worse mental health and problematic anger (Table 4), and they had relatively high R^2^. Not being able to go out, reduced ability to earn, and reduced ability to provide food were also significant factors in the regression associated with increased anger. Moreover, living expenses, reduced ability to earn, work being negatively affected, and being distressed by war noise significantly raised the depression score. Furthermore, not being able to go out and changing the place of residence due to war significantly raised anxiety scores. Finally, not being able to go out and changing the place of residence due to war were significantly associated with increased stress scores.

Although healthcare workers are at the frontline against COVID-19^9^, doctors did not have statistically significantly different anger scores (11.4 ± 4.2), when compared with the mean score of the entire population (11.8 ± 4.3) (Figure 2). They did, however, suffer statistically significantly more from a lower psychological burden on the DASS 21 (42.1 ± 26.2, compared with the overall sample, which had 46.8 ± 27.1). This may be due to a lower number of patients attending emergency departments^26^. However, pharmacists and other healthcare workers had similar anger scores to those of the general population, but higher depression, anxiety, and stress scores, which indicate a higher psychological burden. This was similar to another study that also used the DASS-21. It found that nonmedical healthcare workers, when compared with medical healthcare workers, were at a highest risk of psychological distress from COVID-19^27^.

A study found that a forced nationwide lockdown due to COVID-19 produced panic, anxiety, depression, and PTSD in the long-run^28^. In contrast, implementation of the proper measures prevented additional PTSD symptoms, stress, anxiety, depression, and insomnia when people returned to work^29^. In our study, although anger, depression, anxiety, and stress were associated with difficulties in obtaining personal protection, they were not associated with increased use of personal protection against COVID-19.

Obtaining adequate supplies was among the major sources of worry and one of the main causes of anxiety and anger. This effect continues even after several months of quarantine^8^. In Spain, more than 25% of the studied sample, which was collected using a non-probabilistic snowball, had depression, anxiety, and stress, and the prevalence increased as lockdown progressed^30^. Another study in Nepal, which used online surveys similar to that in our study, found that 84.5% of participants suffered from moderate to severe stress, 11.3% from moderate to severe anxiety, and 7% from moderate to severe depression. Moreover, 25.4% expressed moderate or more severe anxiety disorder which was, along with depression, twice as frequent as reported in the general population^31^. In India, the prevalence of depression increased by 14.4%, anxiety by 8.4%, and stress by 2.9%, after the third week of mandatory lockdown, indicating that there was an of 8–10-fold increase in depression and anxiety^32^. Our study found that anger, depression, stress, and anxiety were highly prevalent after the lockdown due to the collapsed economy and that this effect was greater than the direct effect of the pandemic. Therefore, we advise that future plans not include a full lockdown in fragile countries without proper preparation or strong humanitarian support, as it will lead to a full economic breakdown, and it will only delay the rise in COVID-19 cases without solving the problem.

### Limitations

This study used estimation scales and self-reported measures as this method is easier than fact-to-face interviews, can be widely distributed in a short time and requires few resources. However, more accurate measures should be used with a broader population sample. Furthermore, online and self-reporting methods tend to overestimate symptoms. The SES estimator was also not valid, as discussed in the method section. Furthermore, as the young tend to use the internet more often, the research sample mostly consisted of young people. The requirement for internet access also made it difficult for people who might be severely affected to participate in this research. A higher proportion of female than male participants has been observed in many studies that used the same method in Syria; however, we cannot confirm whether this is because there are more females than males or because they tend to respond to online questionnaires more often. As the research concerns mental health, healthcare workers were more interested in filling out the form and thus comprised a high proportion of the sample, particularly since mental health is severely stigmatized in the Syrian community. The numbers of each profession and their scores can be found in Figure 2. Finally, most of the studied population were from major cities in Syria (Figure 1), and the results might have been skewed, given that the unemployed category comprised the majority of the sample.

All the previous limitations suggest that this study probably underestimated the true burden of mental health disorders among the Syrian population and might affect the generalisability of the study.

## Conclusion

The forced lockdown in Syria had a devastating impact on the economy, which in turn affected the mental health of the population far more than the impact of nine years of war. The psychological burden was enormous, and urgent aid is required as the prevalence of depression is now at its highest, and financial needs are increasing. Household expenses and a reduced ability to obtain medications were identified as major stressors after the lockdown. Not being able to go out, a reduced ability to earn income, increasing living expenses, and difficulty providing food were also among the stressors that had a higher impact on stress, anxiety, anger, and depression. Nonmedical health care personnel were associated with higher psychological distress than doctors. Doctors had a lower psychological burden than others, whereas anger scores were approximately equal regardless of the type of work. Although anger, depression, anxiety, and stress were associated with difficulties in obtaining personal protection, they were not associated with using more personal protection from COVID-19. Furthermore, regular shisha smoking was also associated with more negative feelings, in a similar manner to cigarette smoking.

This study suggests that solutions are required other than the forced lockdown, which had only a temporary effect on reducing the spread of COVID-19. The full lockdown had a devastating effect by destroying the very fragile economy and imposing a heavy psychological burden that may cause more harm than the disease itself. Humanitarian aid is urgently required before it is too late to alleviate the suffering of the people as the psychological impact will take years, if not decades, to resolve.

### Abbreviations

[Table tbl5]

### Declarations

#### Ethics approval and consent to participate

Our study protocol and ethical aspects were approved by Damascus University Deanship, Faculty of Medicine. The approval reference number is 170453.

Online consent was obtained before proceeding with the survey and for the use and publication of the data. Parental consent was requested before proceeding with participants under the age of 16 years.

#### Consent for publication

Online consent for the use and publication of the data was given before participation in the research. Parental consent was requested before proceeding with participants under the age of 16 years.

#### Availability of data and materials

The data can be made available upon reasonable request.

#### Competing interests

We have no conflict of interest to declare.

#### Funding

We received no funding in any form.

### Authors’ contributions

• **AK:** Conceptualization; Data curation; Formal analysis; Investigation; Methodology; Project administration; Supervision; Resources; Validation; original draft; Writing - review & editing.
• **SM:** Investigation; Project administration; Methodology; Resources; Methodology; Writing - review.
• **AG:** Software; Resources; Conceptualization.

All authors have read and approved the manuscript.

### Acknowledgments

We would like to thank Med Dose for their aid in distributing the surveys and disseminating this research work.

## Figures and Tables

**Table 1:** Characteristics of respondents and their mean DAR-5 and DAAS-21 scores

Characteristic	Frequency	Percentage %	Characteristic	Frequency	Percentage %
Gender			Marital Status		
Male	515	35.6	Single	1015	70.3
Female	930	64.4	Engaged	56	3.9
Living in			Married	195	13.5
Countryside	265	18.4	In a relationship	153	10.6
City	1176	81.6	Divorced	17	1.2
Student status			Widowed	7	0.5
No	511	35.5	Smoking cigarette or shisha		
In a secondary school	102	7.1	Not a regular smoker	619	43.0
In a university or college	827	57.4	Regular shisha smoker	183	12.7
Education			Regular cigarette smoker	243	16.9
Elementary school	4	0.3	Smoke both regularly	44	3.1
Until grade 9	16	1.1	Smoker but not regularly	352	24.4
High School	253	17.5	Adequacy of monthly income		
University or college	996	68.9	It cannot cover buying the essentials	337	23.4
Master’s degree or higher	176	12.2	It is only enough for essentials of food and drink	874	60.8
Changed place of residence due to war			It is enough for essentials and other things	227	15.8
No	882	61.9	Dar 5		
Within the same city	230	16.1	Anger not likely	874	62.0
To another city	277	19.4	Anger likely	535	38.0
Yes, I had to do both	37	2.6	Depression		
House that you currently live in			Normal	302	21.8
Owned	953	66.1	Mild	198	14.3
Rented or given by the government	375	26.0	Moderate	318	22.9
Living in friend’s\relative’s house	114	7.9	Severe	202	14.6
Distress from war noises			Very Severe	368	26.5
No	221	15.4	Anxiety		
Yes	1217	84.6	Normal	670	48.3
Life directly endangered from war			Mild	122	8.8
No	176	12.2	Moderate	266	19.2
Yes	978	67.8	Severe	117	8.4
Maybe	288	20.0	Very Severe	213	15.3
Lost someone close due to war			Stress		
No	665	46.2	Normal	399	28.7
Yes	775	53.8	Mild	438	31.6
Chronic medical conditions			Moderate	275	19.8
No	452	68.8	Severe	172	12.4
One chronic disease	187	28.5	Very Severe	104	7.5
Two chronic diseases or more	18	2.7			Std.
Housemates with chronic medical conditions			**Characteristic**	**Mean**	**Deviation**
No	242	21.5	Age	24.83	6.281
One chronic disease	488	43.6	DASS-21	46.67	27.120
Two chronic diseases or more	392	34.9	Depression Score	19.12	11.180
			Anxiety Score	9.84	8.880
			Stress Score	17.72	10.215
			DAR-5 Score	11.81	4.349

**Table 2:** Differences in DAR-5 and DASS-21 mean scores by demographic characteristics

Characteristic		DAR-5			Depression			Anxiety			Stress	
	Mean	Std. Dev.	*P* value	Mean	Std. Dev.	*P* value	Mean	Std. Dev.	*P* value	Mean	Std. Dev.	*P* value
**Gender**												
Male	11.83	4.490	0.893	18.91	11.053	0.618	9.13	8.530	0.027	17.06	10.114	0.078
Female	11.80	4.272		19.23	11.253		10.23	9.045		18.07	10.258	
**Living in**												
Countryside	11.70	4.186	0.654	17.36	10.726	0.006	9.12	8.087	0.164	16.35	9.982	0.021
City	11.84	4.388		19.48	11.227		9.98	9.023		18.00	10.243	
**Student status**												
Not a student	11.53	4.254	0.005	17.83	11.159	0.005	9.27	8.846	0.212	17.30	10.258	0.468
In a secondary school	13.07	4.531		20.76	10.368		10.08	9.358		18.53	9.610	
In a university or college	11.83	4.365		19.69	11.210		10.16	8.852		17.84	10.265	
**Education**												
Elementary	10.75	1.708		18.50	7.550		8.00	6.325		16.00	3.651	
Grade 9	12.56	4.899	0.158	15.87	12.478		6.40	4.290		13.73	9.223	
High School	12.40	4.689		20.69	10.915	0.021	10.88	9.615	0.138	18.81	10.278	0.133
University or college	11.71	4.291		19.13	11.272		9.78	8.685		17.70	10.259	
Master’s degree or higher	11.52	4.102		17.12	10.720		9.10	9.121		16.65	9.942	
**Change in place of residence due to war**												
No	11.66	4.318		18.72	10.985		9.24	8.474		17.27	10.037	
Within the same city	12.08	4.244	0.163	19.81	11.004	0.190	11.50	9.765	< 0.001	18.56	10.257	0.016
To another city	11.86	4.441		19.18	11.436		9.89	8.836		17.81	10.303	
Both	13.08	4.722		22.28	13.833		14.28	10.476		22.28	12.413	
**House that you currently live in**												
Owned	11.75	4.317		19.13	11.108		9.67	8.816		17.69	10.176	
Rented or given by the government	11.80	4.387	0.299	18.75	11.263	0.385	9.78	8.827	0.080	17.35	10.273	0.215
Living in a friend’s\relative’s house	12.43	4.500		20.45	11.484		11.70	9.472		19.31	10.287	
**Distress from war noises**												
No	11.88	4.821	0.814	19.65	11.492	0.444	7.88	8.566	< 0.001	15.82	9.622	0.003
Yes	11.80	4.266		19.01	11.120		10.19	8.865		18.05	10.283	
**Life directly endangered from war**												
No	11.06	4.248	0.007	17.33	11.433	0.013	7.57	8.555	< 0.001	15.40	10.619	< 0.001
Yes	12.05	4.484		19.64	11.225		10.61	9.043		18.54	10.250	
**Lost someone close due to war**												
No	11.78	4.215	0.710	19.28	10.854	0.663	9.38	8.588	0.063	17.58	9.899	0.571
Yes	11.86	4.469		19.02	11.455		10.27	9.123		17.89	10.481	
**Chronic medical conditions**												
No	11.94	4.458	0.443	18.76	10.868		9.27	8.339		17.21	9.873	0.007
One chronic disease	12.32	4.551		20.61	11.693	0.162	12.66	9.859	< 0.001	20.04	11.134	
Two or more chronic diseases	13.00	3.983		20.44	12.935		16.89	11.360		19.56	10.461	
**Housemates with chronic medical conditions**												
No	11.94	4.458		18.76	10.868		9.27	8.339		17.21	9.873	
One chronic disease	12.32	4.551	0.443	20.61	11.693	0.162	12.66	9.859	< 0.001	20.04	11.134	0.007
Two chronic diseases or more	13.00	3.983		20.44	12.935		16.89	11.360		19.56	10.461	
**Marital Status**												
Single	11.79	4.350		19.54	11.048		9.65	8.501		17.66	10.074	
Engaged	12.27	4.602		17.70	12.586		10.48	10.547		17.37	11.537	
Married	11.17	4.098	0.046	15.60	10.479	< 0.001	9.13	9.312	0.154	16.28	9.719	0.042
In a relationship	12.68	4.515		21.21	11.330		11.56	9.960		19.91	11.155	
Divorced	11.56	3.444		21.38	13.099		10.88	9.208		19.75	9.406	
Widowed	10.57	5.062		14.33	11.130		11.67	7.633		16.33	4.274	
**Smoking cigarettes or shisha**												
I am not a regular smoker	11.07	3.968		18.20	10.700		8.98	8.285		16.79	9.721	
Regular shisha smoker	12.67	4.518		19.15	11.591		9.74	8.338		18.33	9.873	
Regular cigarette smoker	12.58	4.525	< 0.001	21.45	12.047	0.002	11.51	10.175	< 0.001	19.52	11.230	0.003
Smoke both regularly	13.59	5.123		21.67	9.657		14.19	9.954		20.24	9.012	
Smoker but not regularly	11.92	4.451		18.80	11.127		9.74	8.779		17.49	10.441	
**Adequacy of monthly income**												
It cannot cover buying the essentials	12.70	4.627		21.07	11.835		11.17	9.694		19.45	11.463	
It is only enough for essentials of food and drink	11.61	4.252	< 0.001	18.94	10.935	< 0.001	9.59	8.648	0.007	17.41	9.936	0.001
It is enough for essentials and other things	11.35	4.137		17.17	10.728		8.97	8.420		16.49	9.023	
Independent t-test and one-way ANOVA were used to determine the significance of the variables in this table.												
DAR-5 maximum score is 20. Anxiety, stress and depression maximum score is 42.												

**Table 3:** Responses to questions regarding COVID-19

Question	Frequency	Percentage %	Question	Frequency	Percentage %
1. Have you or anyone in your household been diagnosed with COVID-19 in the last 2 weeks?			10. How worried have you been in the last 2 weeks that your ability to provide food will be affected?		
No	1376	96.6	Not at all	244	17.6
Yes, myself	2	0.1	Slightly	355	25.6
Yes, a family member	7	0.5	Moderately	334	24.1
Yes, a housemate	26	1.8	Very	454	32.7
2 or more of the above	14	1.0			
2. Have you or anyone in your household been hospitalised in the last 2 weeks?			11. How worried have you been in the last 2 weeks that your ability to obtain medication will be affected?		
No	1249	87.6	Not at all	155	11.2
Yes, myself	16	1.1	Slightly	281	20.3
Yes, a family member	39	2.7	Moderately	305	22.1
Yes, a housemate	45	3.2	Very	640	46.3
2 or more of the above	76	5.3
3. Have you or anyone in your household been asked to self-quarantine with symptoms in the last 2 weeks?			12. How worried have you been in the last 2 weeks that your household expenses will be affected?		
No	1368	96.0	Not at all	128	9.2
Yes, myself	3	0.2	Slightly	273	19.7
Yes, a family member	6	0.4	Moderately	337	24.3
Yes, a housemate	27	1.9	Very	648	46.8
2 or more of the above	21	1.5
4. Have you or anyone in your household been asked to self-quarantine without symptoms in the last 2 weeks?			13. How worried have you been in the last 2 weeks that going out will be affected?		
No	1358	95.3	Not at all	437	31.7
Yes, myself	5	0.4	Slightly	309	22.4
Yes, a family member	7	0.5	Moderately	245	17.8
Yes, a housemate	29	2.0	Very		
2 or more of the above	26	1.8
5. Have you or anyone in your household experienced a reduced ability to earn income in the last 2 weeks?			14. How worried have you been in the last 2 weeks that your living expenses will be affected?		
No	900	63.2	Not at all	38	2.7
Yes, myself	116	8.1	Slightly	103	7.4
Yes, a family member	94	6.6	Moderately	327	23.4
Yes, a housemate	67	4.7	Very	93	66.6
2 or more of the above	248	17.4
6. How worried have you been in the last 2 weeks about becoming infected?			15. How worried have you been in the last 2 weeks that you will become infected from the traffic and crowds?		
Not at all	361	25.7	Not at all	114	8.1
Slightly	593	42.2	Slightly	233	16.6
Moderately	316	22.5	Moderately	403	28.8
Very	136	9.7	Very	650	46.4
7. How worried have you been in the last 2 weeks about friends or family becoming infected?			16. How would you rate the availability of personal protection against COVID-19?		
Not at all	111	7.9	I did not use any of them	241	16.9
Slightly	345	24.4	I found them easy to obtain	500	35.0
Moderately	364	25.8	I found it difficult to buy hand sensitisers	155	10.9
Very	592	41.9	I found it difficult to obtain gloves and face masks	243	17.0
			I found it difficult to buy all of the above	288	20.2
8. How worried have you been in the last 2 weeks that your work will be negatively affected?			17. How committed are you to personal protection?		
Not at all	412	31.3	I wear masks	18	1.2
Slightly	303	23.0	I regularly wash my hands	119	8.2
Moderately	263	20.0	I avoid shaking hands with anyone	9	0.6
Very	340	25.8	I avoid crowds	46	3.2
9. How worried have you been in the last 2 weeks that your studies will be negatively affected?			I avoid seeing people	7	0.5
Not at all	453	35.1	I avoid seeing people	7	0.5
Slightly	235	18.2	I do not do any of the above	152	10.5
Moderately	241	18.7	I do two of the above	276	19.1
Very	361	28.0	I do three of the above	816	56.5

**Table 4:** Differences in DASS-21 and DAR 5 scores according to COVID-19-related responses

Question		DAR-5			Depression			Anxiety			Stress	
	Mean	Std. Dev.	*P* value	Mean	Std. Dev.	*P* value	Mean	Std. Dev.	*P* value	Mean	Std. Dev.	*P* value
**Did any of the following occur because of COVID-19:**												
**1) Hospitalisation**												
No	11.84	4.366		19.16	11.158		9.68	8.841		17.62	10.197	
Yes, myself	13.00	4.676	0.836	19.33	10.438	0.991	12.27	8.447	0.060	23.47	12.839	0.149
Yes, a family member	11.71	4.544		18.31	11.300		10.41	8.946		17.13	11.524	
Yes, a housemate	11.84	3.855		19.55	10.704		10.32	8.092		17.64	8.061	
Two or more of the above	11.57	4.409		19.12	12.744		12.61	9.940		19.31	10.570	
**2) Self-quarantine with symptoms**												
No	11.85	4.357		19.15	11.190		9.90	8.911		17.83	10.267	
Yes, myself	13.33	4.041	0.799	21.33	16.042	0.905	6.67	1.155	0.939	13.33	13.013	0.587
Yes, a family member	10.17	4.215		15.00	10.640		8.67	3.724		12.67	6.890	
Yes, a housemate	12.07	3.970		19.78	11.862		10.59	9.295		17.41	10.150	
Two or more of the above	11.29	5.130		18.86	12.467		10.57	9.448		15.90	8.999	
**3) Self-quarantine without symptoms**												
No	11.79	4.346		19.14	11.209		9.91	8.933		17.76	10.324	
Yes, myself	11.80	3.347	0.459	19.60	12.116	0.898	7.60	5.177	0.981	18.40	8.532	1.000
Yes, a family member	13.00	3.742		23.67	13.589		10.67	5.007		17.00	5.762	
Yes, a housemate	13.10	3.958		18.57	10.312		9.71	8.619		17.86	8.960	
Two or more of the above	12.52	5.561		18.85	12.473		10.15	8.979		17.62	8.617	
**4) Reduced ability to work**												
No	11.72	4.340		18.83	11.161		9.69	8.819		17.60	10.247	
Yes, myself	12.42	4.572	0.002	19.71	11.873	0.001	10.66	9.688	0.025	16.84	10.503	< 0.001
Yes, a family member	10.62	3.551		16.51	10.707		8.26	7.878		14.92	8.591	
Yes, a housemate	11.45	4.039		17.26	9.491		8.74	7.734		16.74	8.599	
Two or more of the above	12.58	4.551		21.45	11.381		11.25	9.291		20.07	10.663	
**5) Was anyone in your household diagnosed with COVID-19?**												
No	11.81	4.350		19.05	11.158		9.85	8.897		17.74	10.228	
Yes, myself	11.50	3.536	0.732	16.00	14.142	0.463	4.00	2.828	0.426	8.00	11.314	0.652
Yes, a family member	13.43	4.315		24.29	14.349		9.43	4.721		17.14	10.511	
Yes, a housemate	12.52	4.483		22.08	12.693		11.69	9.063		19.31	10.968	
Two or more of the above	12.62	5.253		20.14	12.440		13.14	10.280		18.14	10.091	
**6) How worried have you been about being infected?**												
Not at all	11.65	4.753		19.56	11.399		9.60	9.489		17.08	10.710	
Slightly	11.36	4.001	< 0.001	18.05	11.179	0.004	9.19	8.434	< 0.001	16.57	9.782	< 0.001
Moderately	12.17	4.029		19.59	10.714		10.05	7.951		18.63	9.834	
Very	13.47	4.768		21.71	11.298		13.39	10.305		22.39	10.103	
**7) How worried have you been about friends or family being infected?**												
Not at all	11.20	4.548		17.71	11.093		7.65	8.099		15.21	10.077	
Slightly	11.02	4.208	< 0.001	17.55	10.835	< 0.001	8.91	8.703	< 0.001	15.82	9.903	< 0.001
Moderately	11.31	3.958		18.24	10.729		8.83	7.806		16.60	9.552	
Very	12.79	4.461		20.92	11.500		11.54	9.496		20.08	10.433	
**8) How worried have you been that your work will be negatively affected?**												
Not at all	11.53	4.473		17.92	10.949		8.93	8.501		16.22	10.159	
Slightly	11.46	4.036	< 0.001	17.49	10.376	< 0.001	9.44	8.686	0.001	16.37	9.762	< 0.001
Moderately	11.74	4.021		19.19	10.044		10.08	7.984		17.84	8.935	
Very	12.82	4.514		22.39	12.094		11.55	10.097		20.95	11.084	
**9) How worried have you been that your studies will be negatively affected?**												
Not at all	11.43	4.388		18.61	11.569		9.12	8.730		16.32	10.117	
Slightly	11.07	4.134	< 0.001	17.48	10.092	< 0.001	8.30	8.204	< 0.001	15.33	9.502	< 0.001
Moderately	12.18	4.120		18.89	10.389		9.98	8.070		18.42	9.371	
Very	12.79	4.508		21.66	11.521		11.80	9.483		20.64	10.608	
**10) How worried have you been that your ability to provide food will be affected?**												
Not at all	10.88	4.174		15.96	10.124		7.67	7.175		14.38	8.835	
Slightly	11.14	4.218	< 0.001	17.39	10.226	< 0.001	8.77	8.506	< 0.001	15.98	9.401	< 0.001
Moderately	11.82	4.137		19.42	10.866		9.86	8.157		17.96	10.080	
Very	13.04	4.455		22.30	11.924		12.02	9.962		20.92	10.766	
**11) How worried have you been that your ability to obtain medication will be affected?**												
Not at all	10.52	4.121		14.82	10.344		5.73	5.925		12.44	8.545	
Slightly	10.91	4.097	< 0.001	16.22	9.978	< 0.001	7.48	7.535	< 0.001	15.08	9.235	< 0.001
Moderately	11.72	4.333		19.18	10.549		10.75	8.893		17.99	9.509	
Very	12.69	4.345		21.69	11.490		11.58	9.418		20.21	10.575	
**12) How worried have you been that your household expenses will be affected?**												
Not at all	10.37	3.933		14.36	9.940		6.61	6.835		13.16	8.425	
Slightly	10.65	4.103	< 0.001	15.64	9.668	< 0.001	8.05	7.931	< 0.001	15.00	8.958	< 0.001
Moderately	11.55	4.050		18.28	10.139		8.84	7.535		16.31	8.848	
Very	12.80	4.441		22.15	11.670		11.84	9.748		20.60	10.871	
**13) How worried have you been that going out will be affected?**												
Not at all	11.28	4.295		18.01	10.920		8.88	8.696		15.87	10.249	
Slightly	11.40	4.104	< 0.001	17.90	10.371	< 0.001	9.08	8.264	< 0.001	16.74	9.490	< 0.001
Moderately	12.07	3.956		20.75	11.354		9.87	8.131		19.04	9.322	
Very	13.32	4.881		21.39	12.138		12.83	10.181		21.26	11.259	
**14) How worried have you been that your living expenses will be affected?**												
Not at all	10.67	4.579		14.51	9.898		7.54	7.014		12.51	8.194	
Slightly	10.39	3.951	< 0.001	14.43	8.881	< 0.001	7.31	7.128	< 0.001	13.31	8.128	< 0.001
Moderately	10.98	4.179		15.91	10.076		8.57	8.274		15.22	9.700	
Very	12.33	4.369		21.04	11.364		10.69	9.207		19.33	10.314	
**15) How worried have you been that you will become infected from the traffic and crowds?**												
Not at all	10.92	4.505		17.00	10.990		7.25	8.059		14.11	9.581	
Slightly	11.15	4.648	< 0.001	18.28	10.958	0.008	8.93	9.220	< 0.001	16.10	10.158	< 0.001
Moderately	11.39	3.907		18.80	10.846		9.57	8.398		16.92	9.792	
Very	12.60	4.392		20.27	11.464		10.98	9.102		19.57	10.311	
**16) Availability of personal protection against COVID-19:**												
I did not use any of them	12.05	4.985		19.35	11.315		9.52	9.515		17.09	10.648	
I found them easy to obtain	11.33	4.131	0.006	17.63	10.693	< 0.001	9.00	8.432	0.002	16.31	9.723	< 0.001
I found it difficult to buy had sensitisers	11.60	3.814		19.35	10.860		9.93	8.562		17.92	9.693	
I found it difficult to obtain gloves and face masks	12.02	4.264		18.78	10.902		9.76	8.112		18.03	10.139	
It was difficult to buy all of the above	12.49	4.468		21.91	11.826		11.75	9.754		20.41	10.599	
**17) Commitment to personal protection:**												
I wear masks	13.00	4.330		19.67	8.765		11.56	6.308		19.11	7.364	
I regularly wash my hands	11.12	4.214		19.37	11.321		8.84	9.050		17.05	10.537	
I avoid shaking hands with anyone	12.38	4.868		23.00	10.254		13.50	12.224		22.50	10.351	
I avoid crowds	12.89	4.313		17.51	10.731		8.68	7.741		17.95	10.261	
I avoid seeing people	12.67	2.422	0.312	14.67	13.837	0.309	6.33	7.528	0.377	19.33	12.879	0.435
I do not do any of the above	12.12	5.005		20.99	11.966		10.61	9.812		16.85	10.840	
I do two of the above	11.85	4.194		18.30	10.979		9.31	8.240		16.89	9.995	
I do three of the above	11.75	4.294		19.09	11.133		10.04	8.963		18.15	10.160	
One-way ANOVA was used to determine the significance of the variables in this table.												
DAR-5 maximum score is 20. Anxiety, stress and depression maximum score is 42.												

**Table tbl5:** 

COVID-19	Coronavirus disease of 2019
CRISIS	The CoRonavIruS Health Impact Survey
DAR	Dimensions of anger reactions
DASS	Depression Anxiety Stress Scale
PTSD	Post-traumatic stress disorder
SES	Socioeconomic status
